# Liquid Biopsies Using Plasma Exosomal Nucleic Acids

**DOI:** 10.18632/oncoscience.478

**Published:** 2019-04-01

**Authors:** Lino Möhrmann, Filip Janku

**Affiliations:** Department of Investigational Cancer Therapeutics (Phase I Clinical Trials Program), The University of Texas MD Anderson Cancer Center, Houston, TX 77030, USA

**Keywords:** exosomes, liquid biopsy, PCR, next-generation sequencing

Liquid biopsies, which represent isolation of tumor nucleic acids from body fluids to assess molecular profile of cancer, are increasingly accepted as a useful approach in cancer diagnostics and treatment. [1] Liquid biopsies include analysis of tumor circulating cell-free DNA (cfDNA), exosomal nucleic acids or DNA isolated from circulating tumor cells. Liquid biopsies offer a minimally-invasive alternative to the molecular testing of tumor tissue, which is usually obtained from surgical therapeutic or diagnostic procedures. PCR-based methods such as digital PCR or BEAMing digital PCR offer high sensitivity; however, the number of molecular alterations that can be tested is often limited.[1-3] In contrast, next-generation sequencing can detect a broad range of molecular alterations; however, it is often at the cost of lower sensitivity. In addition, the next-generation sequencing workflow is more complex and bioinformatics expertise is needed. [1, 4] Applications of liquid biopsies include detection of molecular targets for cancer therapy, assessment of prognosis, assessment of treatment outcomes, dynamic assessment of clonal evolution, assessment of pharmacodynamics endpoints and early detection. Results of molecular testing of plasma cfDNA-based liquid biopsies were found to be largely concordant with results of molecular testing of tumor tissue especially if blood and tumor tissue were collected around the same time. [2, 3, 5-7] In addition, high amount of mutated cfDNA was found to be associated with shorter survival and/or time to treatment failure. [3, 5-7] Tumor cfDNA is released to the circulation from dying cancer cells, which arguably might not fully represent the prevailing cancer biology. Unlike cfDNA, exosomes contain DNA and RNA originating from living cells. [1, 8] Furthermore, in contrast to short fragments of cfDNA exosomes contain full length DNA, which can reduce complexity of molecular testing. In addition, exosomes also have abundance of RNA, which can be a more suitable material than cfDNA for molecular testing of complex alteration such as fusions. Recently, we demonstrated that combined next-generation sequencing-based molecular testing of plasma-derived exosomal RNA and cfDNA for common oncogenic alterations in *BRAF*, *KRAS*, and *EGFR* genes in patients with advanced cancers has high sensitivity (95%) and specificity (100%) compared to standard clinical testing of tumor tissue. [8] These results were comparable to sensitivity of plasma cfDNA testing with droplet digital PCR (92%) and BEAMing digital PCR (97%). In addition, we also demonstrated that the amount of mutated exosomal RNA and cfDNA is predictive of survival, time to treatment failure and response to treatment. Specifically, patients with a low amount of exosomal mutated RNA and cfDNA in samples collected before therapy had longer median survival (11.8 vs. 5.9 months; *P* = 0.006) and time to treatment failure (7.4 vs. 2.3 months; *P* = 0.009) than patients with higher amount of mutated RNA and/or DNA. In addition, patients with a partial response to treatment or stable disease for at least 6 months had a lower amount of mutated exosomal RNA and cfDNA compared to patients with progressive disease or stable disease lasting for less than 6 months (*P* = 0.006). Therefore, we believe that molecular testing of exosomal nucleic acids can be a useful addition to the portfolio of liquid biopsies, which can further expand clinical applications and utility of liquid biopsies.
